# Association of Three-Month Radiographic Union Score for Tibia Fractures (RUST) with Nonunion in Tibial Shaft Fracture Patients

**DOI:** 10.7759/cureus.8314

**Published:** 2020-05-27

**Authors:** Raman Mundi, Daniel Axelrod, Harman Chaudhry, Navdeep Sahota, Diane Heels-Ansdell, Sheila Sprague, Brad Petrisor, Emil Schemitsch, Jason W Busse, Lehana Thabane, Mohit Bhandari

**Affiliations:** 1 Orthopaedic Surgery, University of Toronto, Toronto, CAN; 2 Orthopaedic Surgery, McMaster University, Hamilton, CAN; 3 Health Research Methodology, McMaster University, Hamilton, CAN; 4 Health Research Methodology, Biostatistics, McMaster University, Hamilton, CAN; 5 Centre for Evidence-Based Orthopedics, McMaster University, Hamilton, CAN; 6 Orthopaedics, Hamilton Health Sciences, Hamilton, CAN; 7 Orthopaedic Surgery, Western University, London, CAN

**Keywords:** tibial fracture, non-union, rust

## Abstract

Objectives

Nonunions of tibial shaft fractures have profound implications on patient quality of life and are associated with physical and mental suffering. Radiographic Union Score for Tibia Fractures (RUST) may serve as an important prognostic tool for identifying patients at a high risk of nonunion.

Design

We used data from the Study to Prospectively Evaluate Reamed Intramedullary Nails in Patients with Tibial Fractures (SPRINT) and Fluid Lavage of Open Wounds (FLOW) trials to explore the association of three-month RUST scores with nonunion in patients with tibial shaft fractures treated with intramedullary nailing. We performed a retrospective cohort study nested within two multi-center, randomized controlled trials.

Participants

The patients included in the current study: (1) sustained a tibial shaft fracture and were enrolled in the SPRINT or FLOW randomized trials, (2) had initial operative management with intramedullary nailing, (3) showed radiographic evidence of an unhealed fracture at the three-month follow-up, and (4) their healing status (union or nonunion) was captured at 12-months postoperatively.

Intervention

Multivariable binary logistic regression was carried out to identify factors associated with nonunion, including open versus closed injury, fracture severity, fracture gap, and three-month RUST score. We determined the concordance statistic (c statistic) for our regression model both with and without the RUST score.

Outcome Measurements and Results

Of the 155 tibial fracture patients with complete data available for analysis, the overall rate of nonunion at 12 months was 30% (n=47). The mean three-month RUST score in patients with nonunion at 12 months was 4.8 (standard deviation (SD) 1.1) as compared to 6.3 (SD 1.7) for those healed at 12 months. In our multivariable regression analysis, open fractures conferred five-fold greater odds of nonunion at 12 months as compared to closed fractures (odds ratio (OR) 4.76, 95% confidence interval (CI):1.71-13.30). Further, three-month RUST scores of 4 and 5-6 were associated with a 47% (95% CI: 18%-73%) and 23% (4.5-51.5%) absolute risk increase of nonunion as compared to a score of ≥ 7, respectively. The addition of RUST scores to our adjusted regression model improved the c statistic from 0.70 (95%CI: 0.61-0.79) to 0.81 (95%CI: 0.74-0.88).

Conclusion

A third of patients with tibial shaft fractures who have failed to heal by three months will show nonunion at one year. Open fractures and lower three-month RUST scores are strongly associated with a higher risk of nonunion at one year. Further research is needed to establish whether prognosis in this high-risk group can be modified.

## Introduction

Tibial shaft fractures represent the most common major long bone fracture surgically treated in the United States, with an annual incidence of 17 per 100,000 people in the developed world [1-2]. Despite a decreasing incidence in developed countries, the overall global incidence of these injuries remains on the rise in direct correlation to increasing rates of road traffic accidents in the developing world [2-3].

It has been estimated that nearly one in every five patients with a tibial shaft fracture will ultimately fail to heal, with profound implications [4]. Patients with nonunion experience significant pain, delayed return to work, physical disability, and mental suffering [5-6]. Furthermore, patients with nonunion have worse physical and mental health than patients with congestive heart failure or myocardial infarction [6]. The consequences of nonunion are also substantial to health care systems as a whole. These patients require significantly greater inpatient and outpatient care, with the total expenditure associated with one nonunion exceeding the costs of an uneventful healing course more than two-fold [7].

To minimize the rates of nonunion associated with tibial shaft fractures, there has been growing interest in delineating risk factors that allow for the appropriate identification and timely management of susceptible patients. Several variables have been recognized as predictors of nonunion such as smoking, unreamed nailing in closed fractures, high-energy injury mechanisms, less cortical continuity/greater fracture gap, and open fractures [4-5,8-13]. However, current studies have focused primarily on identifying baseline injury and treatment characteristics as prognostic factors while early measures of radiographic healing have been less thoroughly investigated. Given the fundamental role of radiographic follow-up in managing patients with such fractures, identifying a radiographic scoring system with strong prognostic capabilities would offer an appealing method of distinguishing patients at an elevated risk for nonunion.

Radiographic Union Score for Tibia Fractures (RUST) is a reliable radiographic scoring system that was developed to assess the healing status of tibial shaft fractures stabilized with intramedullary nailing [14]. Our objective was to determine whether RUST scores at three-months postoperatively are associated with nonunion at 12 months in a cohort of patients identified from two large, multicenter, randomized controlled trials.

## Materials and methods

Study design and eligibility criteria

This study was designed as a retrospective cohort study nested within two randomized controlled trials (RCTs): the Study to Prospectively Evaluate Reamed Intramedullary Nails in Patients with Tibial Fractures (SPRINT) and the Fluid Lavage of Open Wounds (FLOW) trials.

We included all patients that met the following eligibility criteria: (1) were enrolled in the SPRINT or FLOW trials for a tibial shaft fracture, (2) initial operative management consisted of intramedullary nailing, (3) had available radiographs at the three-month follow-up, which demonstrated an unhealed fracture as determined by the treating physician, and (4) their radiographic healing status was documented at 12-months postoperatively.

SPRINT trial

In brief, SPRINT was a multicenter, international, parallel-group, randomized trial of 1,226 patients comparing reamed versus unreamed intramedullary nailing for tibial shaft fractures. The SPRINT trial consisted of a total of 29 sites across Canada, the United States, and the Netherlands. The study eligibility criteria included skeletally mature patients, with open or closed fractures that were non-pathological and amenable to operative fixation with intramedullary nailing. The SPRINT study protocol was approved by the human subject committees at each participating site (REB #99-077-Research Ethics Board/Institutional Review Boards). The complete study methods and results have been previously published [1,14].

FLOW trial

The FLOW trial is a multi-center, randomized trial of 2,549 patients with open fracture wounds conducted at 41 sites across Canada, the United States, Australia, India, and Norway. The FLOW trial utilized a 2 x 3 factorial design to compare two different types of irrigation solutions (i.e. soap vs. saline), as well as three different degrees of irrigation pressure (i.e. high, low, and very low) in the treatment of open fractures. The eligibility criteria of this study included skeletally mature patients with an open fracture wound of an extremity, which required operative intervention. The FLOW study protocol was approved by the human subject committees at each participating site (REB #08-268-Research Ethics Board/Institutional Review Boards). A detailed trial protocol and study results for FLOW have been previously published [15-16].

Data collection

The following patient demographic, injury, and treatment characteristics were retrieved for all eligible patients from both randomized trials: age, gender, ethnicity, smoking status, diabetes history, non-steroidal anti-inflammatory use, mechanism of injury (high energy vs. low energy), number of injuries, open versus closed fracture, open fracture grade (Gustilo and Anderson classification), fracture pattern, fracture gap (<1 cm vs. ≥1 cm), surgical treatment (reamed vs. unreamed), timing to surgery, and wound closure technique. The fracture pattern was classified as complex (comminuted or segmental) or simple (transverse, spiral, oblique). The wound closure technique was categorized as primary closure (closure of wound at initial surgery), delayed primary closure (closure of wound after initial surgery), or secondary closure (closure of wound through flap/grafting). The reported fracture healing status of patients (bone union vs. nonunion) at 12 months was also recorded. Bone union was determined by individual site investigators based on their radiographic and clinical assessment of patients.

Two expert reviewers in musculoskeletal radiology, who were blind to 12-month outcomes, assigned scores according to the Radiographic Union Score for Tibial fractures (RUST) scale to the three-month radiographs of all patients. The RUST scoring system evaluates radiographic fracture healing based on the bridging of each cortex in two radiographic planes (i.e. anterior-posterior and lateral planes). Each of the four cortices is assigned a score of 1 (fracture line, no callus), 2 (bridging callus with visible fracture line), or 3 (bridging callus with no evidence of fracture line) to produce a cumulative score from 4 to 12, with higher scores indicating greater radiographic healing.

Data analysis

Descriptive statistics were used to summarize patient, injury, surgery, and radiographic characteristics. Multivariable binary logistic regression was carried out to explore the association of the following factors with nonunion at one year: (1) open versus closed injury, (2) fracture pattern (complex vs. simple), (3) fracture gap (<1 cm vs. ≥1 cm), and (4) three-month RUST score (≤4 vs. 5-6 vs. ≥7). We limited our regression model to four independent predictor variables (three dichotomous and one three-level ordinal) based on a preliminary assessment of the event rate (47 cases of nonunion) to guard against over-fitting (>9 events per variable) [17]. The selected independent variables were purposefully chosen based on the objectives of the study (RUST scores) and variables that have been suggested to have an association with nonunion (open fracture, comminuted fracture, fracture gap >1 cm) [4,8,11-12]. The concordance statistic (c statistic) was calculated for the regression model with and without the RUST score, to assess the improvement offered by the RUST score for predicting nonunion. We calculated the absolute risk increase (ARI) for each significant predictor and estimated the baseline risk for non-union at one year through identifying patients without the most predictive risk factors for non-unions in regression analysis. The criterion for statistical significance was set at alpha = 0.05. All analyses were performed using IBM SPSS (Version 21, IBM Corp, Armonk, NY).

## Results

A total of 155 patients with tibia fractures and a 12-month follow-up were identified for inclusion, including 83 patients from the SPRINT trial and 72 patients from the FLOW trial. The overall rate of nonunion at 12 months in this cohort of patients was 30% (n=47). For those patients that did heal by 12 months (n=108), the mean time to bone union was 9.4 months (SD 4.2), with only 24% (n=26) of patients achieving union before six months.

Patients were predominantly male (81%), Caucasian (83%), sustained high-energy trauma (81%), and had multiple injuries (i.e. non-isolated fracture, 58%) (Table 1).

**Table 1:** Patient and injury characteristics *High energy defined as: motor vehicle accident (driver/passenger/pedestrian), motorcycle accident, ATV, crush injury, fall from a height, direct trauma (blunt) Low energy defined as: fall from standing, twist, direct trauma (penetrating) FLOW: Fluid Lavage of Open Wounds; SPRINT: Study to Prospectively Evaluate Reamed Intramedullary Nails in Patients with Tibial Fractures; NSAID: nonsteroidal anti-inflammatory drug; ATV: all-terrain vehicle

Characteristic	Union at 12 months N=108	Nonunion at 12 months N=47	Total N=155
Study FLOW	46 (43%)	26 (55%)	72 (46%)
SPRINT	62 (57%)	21 (45%)	83 (54%)
Age, mean (SD) years	39.0 (16.4)	40.9 (13.1)	39.6 (15.4)
Gender			
Female	20 (19%)	10 (21%)	30 (19%)
Male	88 (81%)	37 (79%)	125 (81%)
Ethnicity			
Caucasian	91 (84%)	37 (79%)	128 (83%)
African-American	4 (4%)	2 (4%)	6 (4%)
Asian	5 (5%)	3 (6%)	8 (5%)
Other (Hispanic, Native, etc.)	8 (7%)	5 (11%)	13 (8%)
Current smoker	35 (32%)	14 (30%)	49 (32%)
Diabetic	5 (5%)	2 (4%)	7 (5%)
NSAID use	5 (5%)	4 (9%)	9 (6%)
Mechanism of injury*			
High energy	82 (76%)	44 (94%)	126 (81%)
Low energy	26 (24%)	3 (6%)	29 (19%)
Isolated injury	51 (47%)	14 (30%)	65 (42%)

Furthermore, patients more commonly sustained an open fracture (67%), had a complex fracture pattern (i.e. comminuted or segmental, 57%), and underwent reamed intramedullary nailing for initial surgical management (65%). A total of 119 patients (77%) in this cohort experienced fracture-related complications that comprised primary outcomes in the SPRINT and FLOW trials. Specifically, patients most commonly underwent bone grafting, implant exchange, intramedullary nail dynamization, and re-operation in response to a surgical site infection (Table 2).

**Table 2:** Fracture and surgical characteristics *Patients could have experienced more than one type of complication. For each specific complication, the number listed is the total number of patients experiencing that given complication. * Autodynamization was an adjudicated event only in the SPRINT trial. IM: intramedullary; IQR: interquartile range; SPRINT: Study to Prospectively Evaluate Reamed Intramedullary Nails in Patients with Tibial Fractures

Characteristic	Union at 12 months N=108	Nonunion at 12 months N=47	Total N=155
Closed Fracture	46 (43%)	6 (13%)	52 (34%)
Open Fracture (all)	62 (57%)	41 (87%)	103 (66%)
Type I	11 (10%)	8 (17%)	19 (12%)
Type II	18 (17%)	13 28%)	31 (20%)
Type IIIA	19 (18%)	12 (26%)	31 (20%)
Type IIIB	14 (13%)	7 (15%)	21 (14%_
Type IIIC	0 (0%)	1 (2%)	1 (1%)
Fracture Pattern Complex (Comminuted, Segmental)	54 (50%)	35 (74%)	89 (57%)
Not Complex (Spiral, Oblique, Transverse)	54 (50%)	12 (26%)	66 (43%)
Fracture Location			
Proximal Diaphysis	8 (7%)	11 (23%)	19 (12%)
Middle Diaphysis	42 (39%)	18 (38%)	60 (39%)
Distal Diaphysis	58 (54%)	18 (38%)	76 (49%)
Method of Fixation Unreamed IM Nail	39 (36%)	15 (32%)	54 (35%)
Reamed IM Nail	69 (64%)	32 (68%)	101 (65%)
Post-Operative Fracture Gap <1 cm	105 (97%)	44 (94%)	149 (96%)
≥1 cm	3 (3%)	3 (6%)	6 (4%)
Time to Surgery Median Hours (IQR)	11.8 (6.5-24.0)	11.0 (6.0-20.7)	11.2 (6.4-21.9)
Wound Coverage			
Primary Closure	35 (32%)	20 (43%)	55 (35%)
Delayed Primary Closure	8 (7%)	6 (13%)	14 (9%)
Secondary Closure	19 (18%)	15 (32%)	34 (22%)
Closed Fracture	46 (43%)	6 (13%)	52 (34%)
Fracture Complications	78 (72%)	41 (87%)	119 (77%)
Surgery for Infection*	22 (20%)	8 (17%)	30 (19%)
Bone Graft*	2 (2%)	11 (23%)	13 (8%)
Implant Exchange*	16 (15%)	21 (45%)	37 (24%)
IM Nail Dynamization*	18 (17%)	11 (23%)	29 (19%)
Autodynamization*	27 (25%)	4 (9%)	31 (20%)
Other*	5 (5%)	1 (2%)	6 (4%)

The three-month radiographs were performed at a mean of 92 days after initial surgery. The mean RUST score at the three-month follow-up was 5.9 (SD 1.7).

Prognostic factor comparison: nonunion vs. union

**Figure 1:**
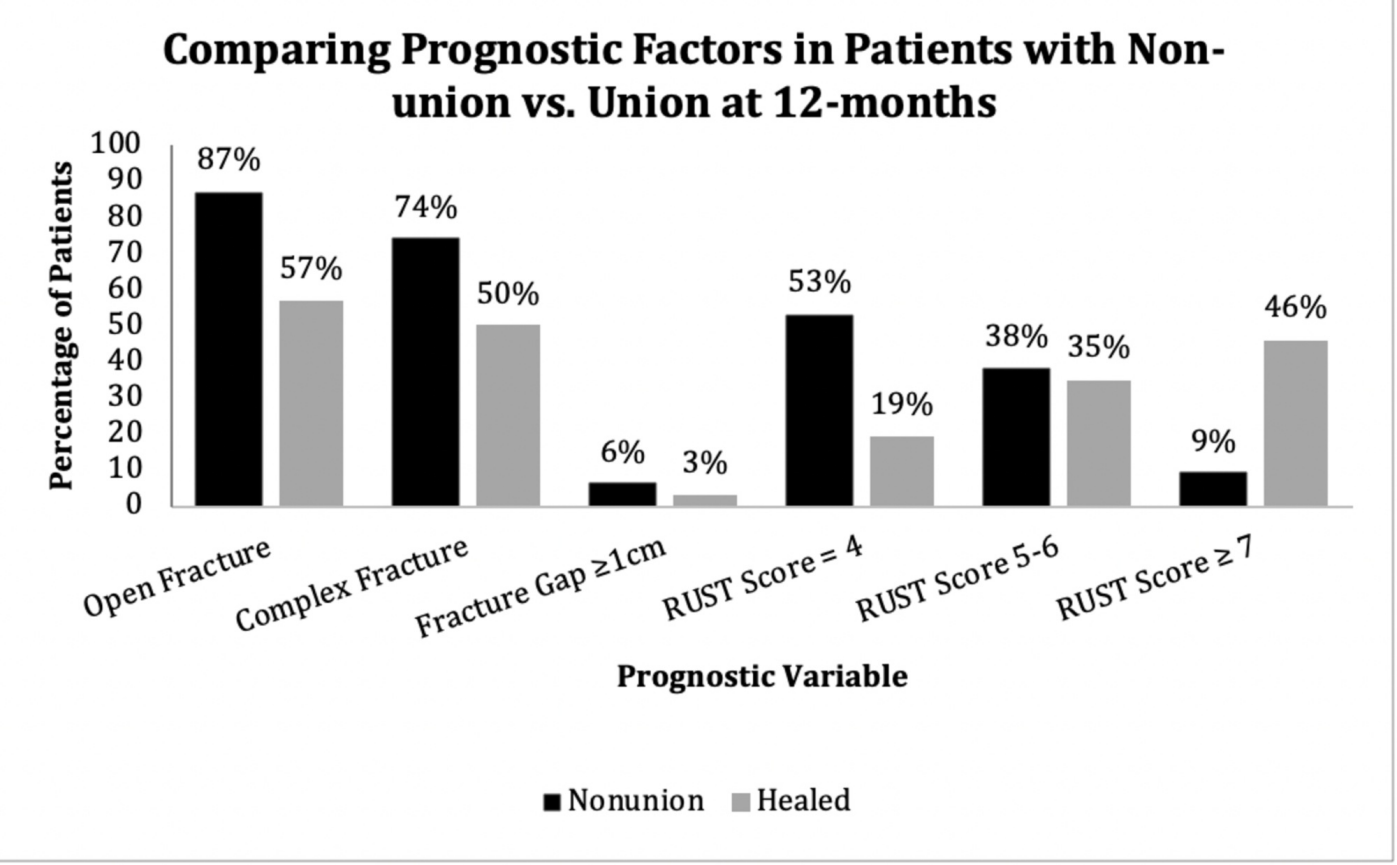
Comparison of prognostic variables between patients with nonunion and union

When comparing patients with nonunion to those healed at 12 months, 87% compared to 57% had an open fracture and 74% compared to 50% had a complex fracture pattern, respectively. Among patients with nonunion, three had a fracture gap of ≥1 cm after initial surgical management (6%, 3/47). Although there were also three patients who went on to heal at 12 months, with a fracture gap of ≥1 cm, this accounted for only 3% of such patients (3/108). The mean three-month RUST score in patients with nonunion was 4.8 (SD 1.1) as compared to 6.3 (SD 1.7) in those healed at 12 months (Figure 1).

Fifty-three percent of patients with nonunion had RUST scores of 4, whereas only 19% of patients healed at 12 months had such low scores (Table 3).

**Table 3:** Three-Month RUST scores in patients with adjudicated adverse events RUST: Radiographic Union Score for Tibia Fractures: SD: standard deviation

Characteristic	Union at 12 months N=108	Nonunion at 12 months N=47
Time to 3-month X-ray mean days (SD)	92.0 (16.8)	91.6 (15.9)
3-month RUST score		
4	20 (19%)	25 (53%)
5-6	38 (35%)	18 (38%)
7-12	50 (46%)	4 (9%)
RUST score, mean (SD)	6.3 (1.7)	4.8 (1.1)

When exploring these risk factors (open versus closed, fracture pattern, fracture gap, three-month RUST score) in a multivariable logistic regression model, only open fracture (odds ratio (OR) 4.8, 95% confidence interval (CI) 1.7 to 13.3, p=0.003) and three-month RUST scores (p<0.001) were found to be associated with nonunion at one year. Compared to a RUST score of ≥7, a three-month RUST score of 4 was associated with 15.5 times greater odds of nonunion (95% CI 4.4 to 54.3); a score of 5 or 6 was associated with 5.7 times greater odds of nonunion (95% CI 1.7 to 18.8). When compared to patients with lowest predicted risk for non-union (RUST7 or higher), patients with RUST scores of 4 or 5/6 had an absolute risk increase for non-union (ARI) of 46.8% (95% CI: 18 - 73.4%) and 23.0% (95% CI: 4.5 - 51.5%), respectively. Fracture pattern and fracture gap ≥1 cm were not significantly associated with nonunion in our analysis (Table 4).

**Table 4:** Multivariable logistic regression (nonunion at 12-months as outcome, N=155) *ARI (95%CI): derived from OR (95%CI) and baseline risk of 7% among the lowest risk population ARI: absolute risk increase; RUST: Radiographic Union Score for Tibia Fractures

Predictor Variable	OR (95% CI)	P-value	ARI (95%CI)*
Open fracture	4.76 (1.71, 13.30)	0.003	19.4% (4.4%, 43.0%)
Complex fracture (comminuted or segmental)	1.46 (0.60, 3.54)	0.401	2.9% (-2.7%, 14.0%)
Fracture gap ≥1cm	0.57 (0.09, 3.46)	0.540	-2.9% (-0.7%, 13.7%)
3-month RUST score			
4	15.49 (4.42, 54.33)	0.001	46.8% (18.0%, 73.4%)
5-6	5.70 (1.73, 18.75)	0.001	23.0% (4.5%, 51.5%)
7-12	1.00		

The addition of RUST scores improved the c statistic for our regression model from 0.70 (95%CI: 0.61-0.79) to 0.81 (95%CI: 0.74-0.88).

## Discussion

Nonunions of tibial shaft fractures have a devastating impact on patient quality of life and often necessitate extensive treatment with unplanned surgical interventions. Approximately one in every three patients with a tibia shaft fracture who have yet to heal at three months and experience a major orthopedic complication related to their fracture will remain unhealed at one year. In the present study, nearly 80% of these patients underwent re-operations to promote fracture healing, including bone grafting, implant exchange, and dynamization. Despite such intervention, the three-month RUST score remained highly effective in the early identification of those patients who remained at risk for nonunion. Patients with little or no callus at three-months (RUST scores of 6 or less), had a 23%-48% absolute risk increase of remaining unhealed as compared to patients with higher RUST scores (≥7). Consistent with previous reports, our study also found a strong association between nonunion and open fractures among tibial fracture patients [8,12].

There has been recent recognition that simple radiographic assessments can be used to successfully predict eventual nonunion in patients with tibial shaft fractures. Yang et al. demonstrated that fellowship-trained trauma surgeons can predict nonunion with 74% accuracy when presented with three-month radiographs in the context of the patient’s clinical scenario [18]. Predictions in this study were largely based on the degree of callus formation and mechanism of injury [18]. In a retrospective review of 176 open and closed tibial fractures treated with intramedullary nailing, Lack and colleagues found that any cortical bridging within four-months postoperatively was strongly predictive of eventual union with 99% accuracy [19]. Of note, this predictive model was based on patients who were simply observed beyond 12 months and did not undergo any unplanned operative interventions to promote fracture healing.

Since the RUST score was first introduced in 2010, multiple studies have demonstrated it to have excellent intra- and inter-rater reliability for assessing the healing status of tibial shaft fractures treated by intramedullary nailing [20-22]. The RUST score has also been shown to correlate with clinical outcomes, including weight-bearing status, patient-reported functional recovery, and the short form-36 physical component score [22-23]. There has been less focus, however, on the prognostic utility of early RUST scores in predicting long-term fracture healing.

In a retrospective analysis of 323 tibia fractures by Ross et al., a bivariate logistic regression was undertaken to evaluate the relationship between findings at the six-week follow-up and eventual tibial non-union. The study determined that four predictors at six weeks (presence/absence of infection, RUST score, modified RUST score, and Non-union Risk Determination Score (NURD)), could generate a prediction model with both sensitivity and specificity for non-union of 82%. However, Ross et al. utilized 40 variable inputs for 50 total non-union events, potentially over-fitting their model to their patient population [24].

More recently, Christiano et al. demonstrated the utility of the RUST score in patients with aseptic tibial nonunion undergoing reoperation to promote healing. Amongst 68 patients presenting, a RUST score of less than 7 predicted persistent non-union even after secondary surgery (sensitivity 1, specificity 0.75) [25]. Lastly, a recent review by our group identified that functional status was a reliable predictor of tibial union [26].

There are several strengths to our study. First and foremost, our patients were part of a randomized controlled trial, in which the surgical procedure and postoperative care were standardized, which limits the impact that these variables may have had on non-union. Furthermore, our conclusions regarding the prognostic utility of the three-month RUST score are derived from a multivariable logistic model that controlled for several confounding variables predictive of nonunion, including open fractures, fracture gap, and fracture pattern (comminuted/segmental). The primary limitation of this study was its retrospective nature. Specifically, three-month radiographs were primarily only available for patients with fracture-related complications, as these adverse events constituted primary outcomes in the SPRINT and FLOW trials that triggered the collection of three-month radiographs. This over-representation of patients with complications provides the likely explanation for a nonunion rate that is higher than most previous reports. However, the capacity of the RUST score to give consistent findings across distinct patient populations, as in this study and the abovementioned studies by Ross and Christiano, underscores the robustness of the three-month RUST score as a prognostic tool for predicting nonunion [24]. Finally, our analysis was limited to the abovementioned four-predictor variables to prevent the over-fitting of our regression model.

## Conclusions

In conclusion, patients with fractures of the tibial diaphysis who experience healing complications are at considerable risk for nonunion at one year following their injury. Both three-month RUST scores and open fractures serve as strong early prognostic indicators of poor healing potential in this population. Future studies evaluating both the efficacy of a timely intervention in improving union rates and identifying new approaches to improve the prognosis for this patient population are warranted.
